# Incremental Learning of Human Activities in Smart Homes

**DOI:** 10.3390/s22218458

**Published:** 2022-11-03

**Authors:** Sook-Ling Chua, Lee Kien Foo, Hans W. Guesgen, Stephen Marsland

**Affiliations:** 1 Faculty of Computing and Informatics, Multimedia University, Persiaran Multimedia, Cyberjaya 63100, Malaysia; 2 School of Mathematical and Computational Sciences, Massey University, Palmerston North 4442, New Zealand; 3 School of Mathematics and Statistics, Victoria University of Wellington, Wellington 6140, New Zealand

**Keywords:** incremental learning, prediction by partial matching, novelty detection, activity recognition, smart homes

## Abstract

Sensor-based human activity recognition has been extensively studied. Systems learn from a set of training samples to classify actions into a pre-defined set of ground truth activities. However, human behaviours vary over time, and so a recognition system should ideally be able to continuously learn and adapt, while retaining the knowledge of previously learned activities, and without failing to highlight novel, and therefore potentially risky, behaviours. In this paper, we propose a method based on compression that can incrementally learn new behaviours, while retaining prior knowledge. Evaluation was conducted on three publicly available smart home datasets.

## 1. Introduction

Many of the countries in the world are experiencing growth in terms of the proportion of older adults in the population. In 2020, there were 727 million people aged 65 years or over, and it is projected that the number of older adults will double to 1.5 billion in 2050 [1], representing the fastest growing segment of the world’s population. Enabling people to age independently in their own homes is clearly necessary both for their wellbeing and to avoid a caregiver crisis.

Advances in pervasive computing and wireless sensor networks have resulted in the development of monitoring systems such as smart homes. A variety of unobtrusive sensors such as binary and motion sensors are installed in a smart home to collect information about the inhabitant. These sensors, which record the inhabitant’s interactions within the home (e.g., turning on the light, opening the bathroom door) are used to infer the inhabitant’s daily activities (e.g., showering and cooking). Significant deviations from normality are then detected as potentially risky behaviours, and a query issued.

Many activity recognition systems based on supervised learning have been proposed [2,3,4,5,6]. These systems learn from a set of training data where the activities are labelled a priori, and assume that the inhabitant’s activities remained constant over time. However, human behaviours are rarely so consistent; for example, changes in season may affect sleeping patterns and mealtimes. Systems that do cater for such variability will misclassify the changed patterns, which hinders their utilisation in real homes.

For a smart home to support its inhabitant, the recognition system should not only recognise their activities, but continuously learn and adapt to the inhabitant’s ongoing changing behaviours. The application of novelty detection in learning systems is one of the commonly used methods where the system uses the trained model to learn about inputs that it has never seen before. Some works have attempted to extend novelty detection to learn incrementally by retraining when a previously unseen activity is detected [7,8,9]. However, this is a significant computational overhead, and may allow the catastrophic forgetting of old behaviours, where the performance of the previously learned activities significantly decreases as new activities are learned.

The central problem that this paper aims to address is *how to identify unseen new activities that were not present in the training data and then learn about recurring new activities without forgetting previously learned ones*. Our approach to this problem is to first train a base model using an adaptive lossless compression scheme based on the prediction by partial matching (PPM) method by exploiting the repetition in the sensor stream, which represents the inhabitant’s activities. This base model is then used to guide the learning of new activities.

The remainder of this paper is organised as follows: Section 2 discusses the related work. Section 3 provides a description of the method used. Section 4 presents our proposed method. Section 5 describes the benchmark datasets used in this study. Section 6 details the experiments and evaluation method. The results and findings are discussed in Section 7. Section 8 provides a summary of our work.

## 2. Related Work

Novelty detection often requires a machine learning system to act as a ‘detector’, which identifies whether an input is part of the data that a machine learning system was trained on. This will result in some form of novelty score, which is then compared with a decision threshold, where new unseen inputs are classified as novel if the threshold is exceeded. Novelty detection has gained much research attention, especially in diagnostic and monitoring systems [10,11,12]. An overview of the existing approaches is provided in [13].

There are works that use the one-class classification approach for novelty detection. In this approach, the classifier is trained with only the normal data, which are then used to predict new data as either normal or outliers [14]. In the work of [15], they extracted nonlinear features from vibration signals and used these features to detect novelty. This method, however, requires an extensive preprocessing step for feature extraction. Rather than applying one-class classification on preprocessed data, Perera and Patel [16] used an external multi-class dataset for feature learning based on a one-class convolutional neural network. Although this method bypasses the data preprocessing step, the performance of such a system is highly dependent on the hyperparameter selection and a large quantity of training data.

Another approach to novelty detection is to use an ensemble [17]. A normality score is computed from the consensus votes obtained from the ensemble models, and a threshold value is dynamically determined based on the distribution of the normality score from each ensemble model in order to identify novelty. This approach, however, does not learn incrementally, nor does it adapt to new activities. In the work of [7], they extended the ensemble approach to allow activities to be learned incrementally. When a new activity is detected, a new base model is trained and is added to a set of previously trained base models. One of the problems with this approach is the increase in the ensemble size when more activities are learned, which can significantly affect the performance of previously learned activities.

To avoid overwriting previously learned activities, Ye and Callus [18] proposed using a neural network to iteratively learn new activities by reusing the information from a previous trained network to train a new network. A gradient-based memory approach is applied to control the update of the model parameters. Although this method is able to maintain the knowledge of previous activities, it is memory-intensive.

A recent method was proposed by [19] for novelty detection. In this method, they first compressed the sensor stream to identify repeated patterns that represent activities. A new activity was identified by monitoring the changes in the frequency distribution. Since patterns have to be repeated frequently in order to generate significant changes in the frequency distributions, this method takes more time to learn a new pattern. A similar work was seen in [20], where they combined the Markov model and prediction by partial matching for route prediction. New routes were detected by measuring the similarity between the original route and predicted route that the user is likely to traverse. The similarity is measured in terms of the rate of compression, which is computed from the partial matching trees and Markov transition probabilities. Although this method is able to predict new routes, it needs prior knowledge of user destinations.

## 3. Prediction by Partial Matching (PPM)

Prediction by partial matching (PPM) is an adaptive statistical data compression technique that uses the last few symbols to predict the next symbol in the input sequence [21]. PPM adaptively builds several *k* context models, where *k* refers to the number of preceding symbols used.

Following the approach taken in [22], the PPM is built based on each activity sequence, *S*, which is represented as a triplet of ASCII characters identifying the time when the activity is performed, the location, and the type of activity: $S_{i}=\langle time,location,activity\rangle$. Given the input string ‘*activeactionick*’, let $S_{1}=\left(a,c,t\right)$, $S_{2}=\left(i,v,e\right)$, $S_{3}=\left(a,c,t\right)$, $S_{4}=\left(i,o,n\right)$, and $S_{5}=\left(i,c,k\right)$. The PPM is trained on each sequence of $S_{i}$ rather than on the entire input string. Table 1 shows the results of three context models with k=2,1, and 0 after the input string ‘*activeactionick*’ has been processed.

With this, the highest context model (k=2) predicts the user’s activity given the time and location (i.e., (time,location)→activity)), while the k=1 model predicts: (1) the user’s location given the time of the day (time→location) and (2) their activity given the location (location→activity).

When the PPM model is queried, the model starts with the largest *k* (here, 2). When the string ‘*io*’ is seen, the likely next symbol is *n*, with a probability of 0.5. If a new symbol is observed in this context, then an escape (‘*esc*’) event is triggered, which indicates a switch to a lower-order model. This process is repeated until the context is matched or the lowest model (k=−1) is reached. The lowest model predicts all symbols equally with $p=\frac{1}{\left|A\right|}$, where *A* is the set of distinct symbols used.

## 4. Proposed Method

The first aim of this paper is to detect novel activities, i.e., activities that were not present during the training of the PPM model. We achieve this by calculating a novelty score that measures how similar the new input is to the learned activities. This novelty score can be computed in terms of compression factor (*CF*), defined as in [23]:

(1) $$CF=\frac{Size\;of\;Uncompressed\;Data}{Size\;of\;Compressed\;Data}$$

The higher the factor, the better the compression, i.e., the more similar the novel input is to the learned activities. To calculate the size of the compressed dataset, our method leverages the *esc* event in the PPM model. The rationale behind this approach is that if an input string contains context similar to the PPM model, the compression process will rarely activate the *esc* event, resulting in a higher *CF*. However, if the input string differs greatly from the PPM model, the *esc* event will be triggered more frequently, resulting in a lower *CF*.

If the input string ‘act’ has been seen frequently in the past, then it is likely to recur identically in the future. However, if there are variations in the input string (suggesting variations in the activities), the next occurrence will be followed by different symbols, e.g., ‘ack’ or ‘ict’. This will trigger the PPM model to switch to a lower model. To determine the size of the compressed and uncompressed data, we calculate the entropy, in units of bits, from the probabilities obtained from the PPM model. Section 4.1 provides further examples of how *CF* is calculated to detect novel activities.

**Figure 1:**
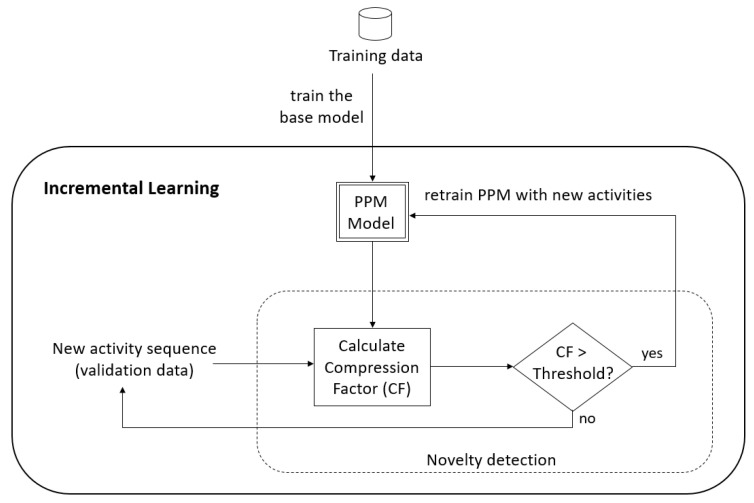
Summary of our proposed method.

One of the challenges in detecting novel activities is that the input pattern could be an entirely new activity that has not been seen before, or it could be just noise in the data. For this, a threshold is applied to quantify the novelty. Novelty is detected when the *CF* value is above the threshold. Figure 1 summarises the overall procedure of the proposed method. Algorithm 1 shows the steps of detecting new activities.
**Algorithm 1** Novelty Detection based on Prediction by Partial Matching (PPM)**Input:** P← base PPM model trained on training set
**Input:** S= activity sequence on validation set
**Initialise:** N={}
**Initialise:** t= threshold value **for** 
i=1,2,…,|S| 
**do**   CF← Using Equation (1), calculate compression factor $\left(P,S_{i}\right)$   **if** CF>t **then**     $N\leftarrow N\cup S_{i}$   **end if** 
**end for** 
P← Retrain *P* with *N*


### 4.1. Detecting Unseen New Activities

Suppose that the PPM model shown in Table 1 is trained from the following input data:

(8 a.m., Kitchen, Preparing Meal) →(a,c,t)
(9.30 a.m., Bathroom, Bathing) →(i,v,e)
(9.30 a.m., Bedroom, Dressing) →(i,o,n)
(9.30 a.m., Kitchen, Washing Dishes) →(i,c,k)

**Figure 2:**
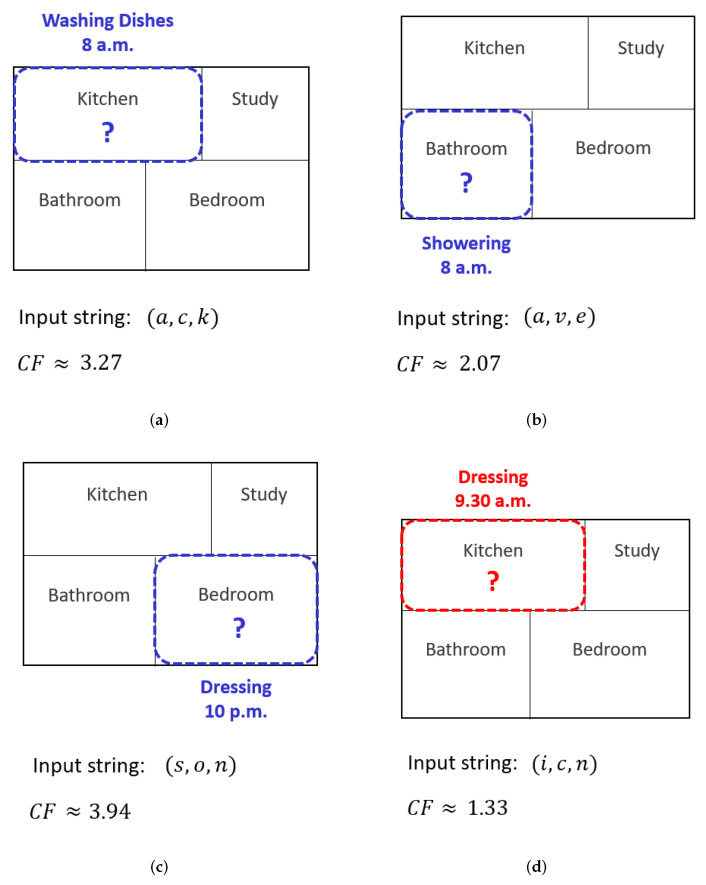
Illustration showing how novelty is detected by calculating the compression factor. (**a**) Similar time and location, different activity. (**b**) Similar location and activity, different time. (**c**) Similar location and activity, new time. (**d**) Similar time and activity, different location. For details, see the text.

Once the PPM is trained from the input string ‘(a,c,t)(i,v,e)(a,c,t)(i,o,n)(i,c,k)’, this base PPM model is used for novelty detection. Given that there are nine distinct characters, the entropy of the uncompressed data is $-log_{2}\left(\frac{1}{9}\cdot \frac{1}{9}\cdot \frac{1}{9}\right)\approx 9.51$ bits. Figure 2 illustrates how *CF* is computed based on four different scenarios. The size of the compressed data is computed based on the PPM model shown in Table 1. If the novelty threshold is 2.0, novelty is detected for the scenarios shown in Figure 2a–c since the *CF* value is above the threshold in those instances.

In the figure, (a) shows an example where a different activity was seen at a similar time and location in the past (i.e., ‘washing dishes’ instead of ‘preparing meal’). When the input string (a,c,k) is detected, the k=2 model is first queried for ac→k. Since the string ‘*ac*’ is seen in the k=2 model, meaning that the prediction of a→c will be in the k=1 model, the *esc* event is triggered to switch to the k=1 model. Both strings ‘*ac*’ and ‘*ck*’ are queried and the size of the compressed data is computed as $-log_{2}\left(P\left(a\to c\right)\cdot P\left(c\to k\right)\right)=-log_{2}\left(\frac{2}{3}\cdot \frac{1}{5}\right)\approx 2.91$ bits. Using Equation (1), the *CF* for the input string (a,c,k) is $\frac{9.51}{2.91}\approx 3.27$. Since the *CF* is above the threshold, novelty is detected.

(b) shows an example where a similar location and activity were seen in the past but at a different time. Since the input string (a,v,e) is not seen in the k=2 model, the *esc* event is triggered to switch to the k=1 model. The string ‘*ve*’ is seen (P(v→e)), but not ‘*av*’. An *esc* event is triggered to switch to k=0 by taking P(a). The size of the compressed data for the input string (a,v,e) is $-log_{2}\left(P\left(v\to e\right)\cdot P\left(a\right)\right)$, with *CF* ≈2.07. Novelty is detected for this input string since the *CF* is above the threshold.

For the input string (s,o,n) in (c), the string ‘*on*’ is seen (P(o→n)) in k=1, but not ‘*so*’. This will trigger the *esc* event to switch to k=0. Since the string ‘*s*’ is a new time and has not been seen before, we take P(esc) to calculate the size of the compressed data ($-log_{2}\left(P\left(o\to n\right)\cdot P\left(esc\right)\right)$). The *CF* for this input string is approximately 3.94 and novelty is detected.

(d) shows an example where a similar activity at a similar time was seen in the past, but the activity was performed in a different location. For the input string (i,c,n), the string ‘*ic*’ is seen in k=1 (P(i→c)). Since the string ‘*cn*’ is not seen, an *esc* event is triggered by taking P(n). The *CF* for this input is approximately 1.33, which is below the threshold and therefore no novelty is detected.

## 5. Data Source

We tested our approach on three publicly available smart home datasets, which we summarise in Table 2. In each of these datasets, the home inhabitant noted their activities, providing ground truth annotations.

## 6. Experiments and Evaluation Method

We evaluated the recognition performance and time required to train the PPM in comparison with other approaches, and also tested the effect of the size of the training dataset on recognition performance. We partitioned the data into training, validation, and testing sets according to the splits shown in Table 3, using 6-fold cross-validation.

**Figure 3:**
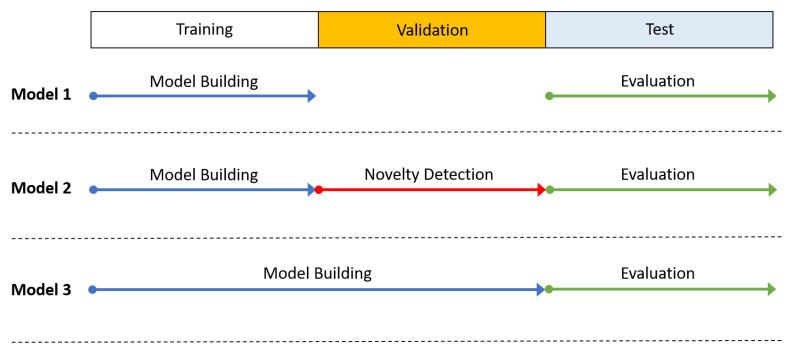
Implementation of the 3 models based on training and validation sets.

Our approach (labelled Model 2) uses the validation set to perform novelty detection. As a comparison, we included a model that does not use the validation data at all (Model 1) and another that is trained on both the training and validation sets following the approach taken in [22]. Both Model 1 and Model 3 are learned from a predefined set of activities and are used as the baseline models. Figure 3 shows the implementation of the three models based on the respective training–validation sets.

To evaluate the effectiveness of our method, three evaluations were carried out. The first evaluates the recognition performance in terms of predicting the user’s location given the time of the day (time→location ). The second evaluates the recognition performance in terms of predicting the user’s activity given the location (location→activity). The first two evaluations use the k=1 context model for prediction. The third evaluates the recognition performance in terms of predicting the user’s activity given location and time ((time,location)→activity). This evaluation used the k=2 context model for prediction.

We also determined the effect of the training dataset size on the base PPM model and the model’s capability for incremental learning by reducing the training and validation sets to 5 days each for the Aruba and van Kasteren datasets, and 3 days each for the MIT PlaceLab dataset. All of the remaining data (48 days for Aruba, 10 for MIT PlaceLab, and 14 for van Kasteren) were used for testing. For this evaluation, 8-fold cross-validation was used.

Finally, we measured the time required to train the PPM using Matlab on a desktop computer with an Intel(R) Core(TM) CPU i7-7700K @ 4.2 GHz and 64 GB memory.

## 7. Results and Discussion

Table 4 shows the recognition performance of time→location and location→activity predictions. The recognition performance of (time,location)→activity prediction is shown in Table 5. In comparison with baseline Model 1, our method (Model 2) achieved a higher performance for time→location (Aruba: 91.41%, MIT PlaceLab: 87.57%, van Kasteren: 80.82%), location→activity (Aruba: 98.73%, MIT: 98.69%, van Kasteren: 99.87%), and (time,location)→activity (Aruba: 88.02%, MIT: 73.87%, van Kasteren: 79.21%) across all of the datasets. The results show that our method is able to incrementally learn new activities and can improve the recognition performance of the baseline model when trained on the same amount of data.

However, when compared with Model 3, we can see that the amount of data matters: our model has a lower, but comparable, performance. However, using Model 3 requires twice as much waiting for activities to appear and be learnt from the data (a time frame of 30 days vs. 15 days). By using our method, we can deploy a baseline model for activity recognition (Model 1), and improve the recognition performance by allowing it to learn new activities when new data are available. This result suggests that a general PPM model can be used as a base model in various smart homes and the recognition performance of this base model can be improved by using our method.

**Figure 4:**
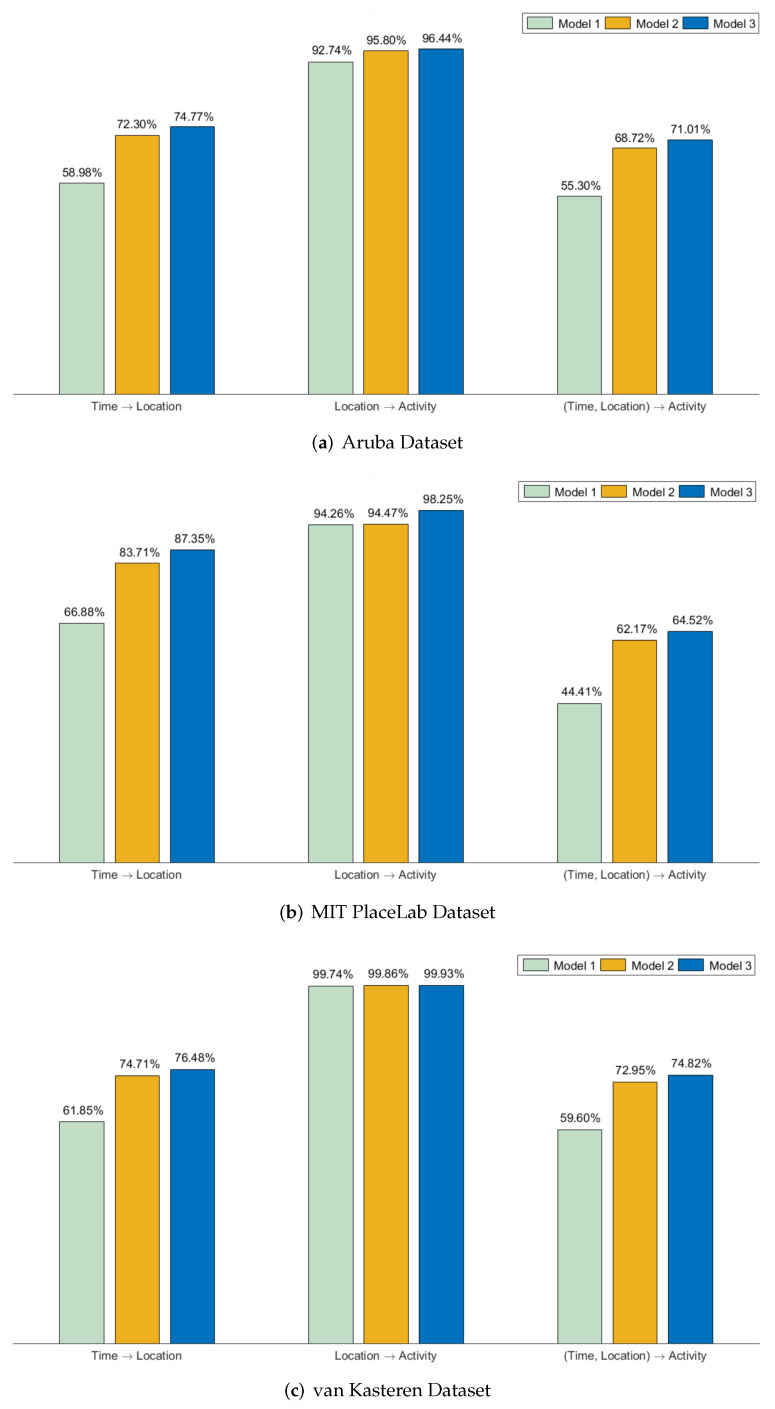
Average recognition accuracy of the 3 models trained on a smaller training set.

Figure 4 shows the results of the three models trained on different training–validation–test splits. In terms of the size of the training dataset, when trained on a smaller training set, Model 1 suffers across all three datasets for time→location and (time,location)→activity, with a performance as low as 44.41%. Model 2 shows an increment of more than 10% across all of the datasets for time→location and (time,location)→activity when compared with Model 1. In terms of the location→activity prediction, Model 1 has a slightly lower performance compared with when it is trained with a larger dataset. However, we can still see that Model 2 shows improvement in the recognition performance. A lower recognition performance was observed for (time,location)→activity compared with location→activity across all three datasets. This was due to the variations in the time at which the user performed the activities. These variations were not repeated frequently enough for the base PPM to learn the representations. Compression tends to be more effective when patterns are repeated frequently. When trained on a smaller training set, the performances of Models 2 and 3 are comparable across all three datasets. These results show that the ability of our method to carry out incremental learning is not affected by the training size. Our method allows the algorithm to continuously learn in order to improve the recognition performance of the base model, even if the base model is trained with a very small training set.

Table 6 shows the amount of time (in minutes) it took to train the PPM for each model. The values in parentheses show the number of activity instances in each training set. As can be seen from Table 6, the training time grows with the number of activity instances. When comparing all three models, Model 3 has the longest training time since it trains on a larger number of activity instances. Model 2, even though it includes the time to retrain the PPM when new activities are detected, has a slightly shorter training time than Model 3. Although the time difference is not significant, Model 2 allows new activities to be incrementally learned when new data are available.

In this study, the threshold used to quantify the novelty was chosen to be 2.0 based on preliminary experiments. However, the threshold could be determined dynamically from the probability distribution of the data. Methods that could potentially be applied include internal and external voting consensus schemes [17].

We also plan to extend our work to monitor potential abnormality. The challenge lies not in the activity itself, but rather when and where the activity actually takes place. We can further extend the use of *CF* score to determine the abnormal activity (as shown in Figure 2d). Our work is currently applied in a batch manner, but can be extended for online learning. Once new activity is detected, the probabilities in the PPM model can be updated directly instead of retraining the entire PPM model.

## 8. Conclusions

The majority of previous studies on activity recognition consider learning in a fixed environment, where the living environment and activities performed remain constant. However, variability is normal; both human activities and the environment can change over time. In this paper, we proposed a method based on prediction by partial matching that has the ability to continuously learn and adapt to changes in a user’s activity patterns. The main advantage of our approach is that new activities can be incrementally learned in an unsupervised manner. Experiments were performed on three distinct smart home datasets. The results demonstrate that our method works effectively to identify new activities, while retaining previously learned activities.

## Figures and Tables

**Table 1:** PPM model showing the three context models with k=2,1, and 0 after processing input string ‘*activeactionick*’. The frequency counts in the column labelled *c* and the probabilities *p* of each symbol are maintained by the model.

k=2					k=1					k=0			
Predictions			*c*	*p*	Predictions			*c*	*p*	Predictions		*c*	*p*
(*Time*,*Location*) → *Activity*					*Time* → *Location*					→	*a*	2	$\frac{2}{24}$
*ac*	→	*t*	2	$\frac{2}{3}$	*a*	→	*c*	2	$\frac{2}{3}$	→	*c*	3	$\frac{3}{24}$
	→	*esc*	1	$\frac{1}{3}$		→	*esc*	1	$\frac{1}{3}$	→	*e*	1	$\frac{1}{24}$
										→	*i*	3	$\frac{3}{24}$
*ic*	→	*k*	1	$\frac{1}{2}$	*i*	→	*c*	1	$\frac{1}{6}$	→	*k*	1	$\frac{1}{24}$
	→	*esc*	1	$\frac{1}{2}$		→	*o*	1	$\frac{1}{6}$	→	*n*	1	$\frac{1}{24}$
						→	*v*	1	$\frac{1}{6}$	→	*o*	1	$\frac{1}{24}$
*io*	→	*n*	1	$\frac{1}{2}$		→	*esc*	3	$\frac{3}{6}$	→	*t*	2	$\frac{2}{24}$
	→	*esc*	1	$\frac{1}{2}$						→	*v*	1	$\frac{2}{24}$
					*Location* → *Activity*					→	*esc*	9	$\frac{9}{24}$
*iv*	→	*e*	1	$\frac{1}{2}$	*c*	→	*k*	1	$\frac{1}{5}$				
	→	*esc*	1	$\frac{1}{2}$		→	*t*	2	$\frac{2}{5}$				
						→	*esc*	2	$\frac{2}{5}$				
					*o*	→	*n*	1	$\frac{1}{2}$				
						→	*esc*	1	$\frac{1}{2}$				
					*v*	→	*e*	1	$\frac{1}{2}$				
						→	*esc*	1	$\frac{1}{2}$				

**Table 2:** Overview of the datasets used in this study.

Description	Aruba [24]	MIT PlaceLab [25]	van Kasteren [26]
Period	58 days	16 days	24 days
Rooms	7	4	4
Activity Instances	7357	1805	1318
Activities	(a) Meal preparation	(a) Grooming/dressing	(a) Toileting/showering
	(b) Eating	(b) Doing/putting away laundry	(b) Going to bed
	(c) Working	(c) Toileting/showering	(c) Preparing meals/beverages
	(d) Sleeping	(d) Cleaning	(d) Returning/leaving house
	(e) Washing dishes	(e) Preparing meals/beverages	
	(f) Bed to toilet	(f) Washing/putting away dishes	

**Table 3:** Partition of training, validation, and test sets.

Dataset	Number of Days						
	**Model 1**		**Model 2**			**Model 3**	
	**Training**	**Test**	**Training**	**Validation**	**Test**	**Training**	**Test**
(a) Aruba	15	28	15	15	28	30	28
(b) MIT PlaceLab	5	6	5	5	6	10	6
(c) van Kasteren	7	10	7	7	10	14	10

**Table 4:** Recognition performance for time→location and location→activity predictions.

Recognition Accuracy (%)						
	time→location			location→activity		
Test Set	Model 1	Model 2	Model 3	Model 1	Model 2	Model 3
(a) Aruba Dataset						
1	90.81	91.44	96.13	100	100	100
2	80.43	93.17	96.13	94.79	100	100
3	94.27	95.90	98.17	100	100	100
4	80.97	95.96	98.17	96	96	100
5	80.76	86.40	85.60	96.52	100	100
6	73.61	85.57	85.60	97.16	99.56	100
**Average**	**83.47**	**91.41**	**93.30**	**97.28**	**98.73**	**100**
(b) MIT PlaceLab Dataset						
1	76.94	82.08	89.31	95.28	95.28	99.17
2	77.22	85.56	94.71	99.17	99.17	99.56
3	79.12	85.00	89.31	98.97	99.56	99.17
4	83.68	86.77	96.33	98.82	99.56	99
5	88.82	91.49	96.33	99	99	99
6	89.82	94.49	94.71	97.16	99.56	99.56
**Average**	**82.60**	**87.57**	**93.45**	**98.07**	**98.69**	**99.24**
(c) van Kasteren Dataset						
1	61.59	67.34	69.71	100	100	100
2	57.70	67.51	69.71	99.32	100	100
3	77.98	90.30	91.92	99.80	99.80	99.80
4	77.78	90.10	91.92	99.80	99.80	99.80
5	77.33	85.00	85.01	99.82	99.82	99.82
6	72.58	84.64	85.01	99.82	99.82	99.82
**Average**	**70.83**	**80.82**	**82.21**	**99.76**	**99.87**	**99.87**

**Table 5:** Recognition performance for (time,location)→activity prediction.

Recognition Accuracy (%)			
Test Set	Model 1	Model 2	Model 3
(a) Aruba Dataset			
1	87.85	88.59	90.38
2	75.02	88.87	90.38
3	92.37	94.50	96.10
4	75.24	91.86	96.10
5	77.58	83.11	82.32
6	68.44	81.19	82.32
**Average**	**79.42**	**88.02**	**89.60**
(b) MIT PlaceLab Dataset			
1	50.83	63.47	70.97
2	55.14	68.89	82.21
3	58.97	72.79	70.97
4	69.12	79.12	80
5	65.78	79.30	80
6	64.61	79.63	82.21
**Average**	**60.74**	**73.87**	**77.73**
(c) van Kasteren Dataset			
1	60.58	66.33	68.02
2	55.84	65.82	68.02
3	76.77	88.69	89.90
4	76.57	88.08	89.90
5	75.69	83.36	83.36
6	71.48	83.00	83.36
**Average**	**69.49**	**79.21**	**80.43**

**Table 6:** Time required for training the PPM. The number of activity instances for each training set is shown in parentheses.

Training Time (In Minutes)			
Training Set	Model 1	Model 2	Model 3
(a) Aruba Dataset			
1	39.74 (2438)	131.30 (3796)	136.06 (3842)
2	9.21 (1404)	106.29 (3549)	136.06 (3842)
3	39.87 (2438)	176.65 (4236)	197.25 (4409)
4	21.07 (1971)	166.41 (4204)	197.25 (4409)
5	15.96 (1733)	109.26 (3608)	117.93 (3704)
6	20.95 (1971)	105.95 (3588)	117.93 (3704)
**Average**	**24.46 (1993)**	**132.64 (3830)**	**150.41 (3985)**
(b) MIT PlaceLab Dataset			
1	0.9975 (536)	4.1370 (985)	5.2030 (1085)
2	1.0289 (549)	4.4180 (1022)	6.0415 (1206)
3	1.0460 (536)	4.1230 (989)	5.2030 (1085)
4	1.3110 (589)	5.4954 (1094)	6.9420 (1125)
5	1.4127 (617)	5.7956 (1131)	6.9420 (1125)
6	1.3168 (589)	6.2595 (1151)	6.0415 (1206)
**Average**	**1.1855 (569)**	**5.0381 (1062)**	**6.0621 (1139)**
(c) van Kasteren Dataset			
1	0.4257 (371)	1.5950 (677)	1.9195 (727)
2	0.3830 (356)	1.6893 (696)	1.9195 (727)
3	0.4301 (371)	1.6576 (686)	2.7627 (823)
4	0.7158 (452)	2.3508 (764)	2.7627 (823)
5	0.3174 (319)	1.5876 (664)	2.3993 (771)
6	0.7248 (452)	2.1285 (729)	2.3993 (771)
**Average**	**0.4995 (387)**	**1.8348 (703)**	**2.3605 (774)**

## Data Availability

Not applicable.
